# Gram's Stain Revisited

**DOI:** 10.1155/S106474499500038X

**Published:** 1995

**Authors:** David E. Soper

**Affiliations:** Department of Gynecology and Obstetrics Medical College of Virginia Richmond Virginia USA


Infectious Diseases in Obstetrics and Gynecology 3:89–90 (1995)

© 1995 Wiley-Liss, Inc.

### Gram's Stain Revisited

Little did Christian Gram[1] know that his differential staining technique developed in 1884 would remain one of the first procedures learned by beginning bacteriology students and one of the first procedures carried out in any laboratory where bacteria are routinely identified at the end of the 20th century. In today's high-tech medical world where the utilization of nucleic-acid amplification is becoming the norm, it is refreshing to know that even the novice health-care provider can perform and interpret this simple test. In fact, this test is arguably underutilized.

The CDC[2] continues to recommend its use in the evaluation of the patient with mucopurulent endocervicitis. We continue to routinely order a Gram's stain of the sputum in patients with lower respiratory infections. A Gram's stain of the urine can be helpful in directing the initial therapy of a patient with pyelonephritis by ruling out the uncommon patient with a gram-positive infection. Many experts suggest that a Gram's stain of culture material gathered from the endometrium in a woman who has failed her initial antimicrobial therapy is helpful in directing changes in antimicrobial chemotherapy. For example, a patient being treated with a cephalosporin who fails to respond to therapy and whose subsequent Gram's stain of the endometrium shows gram-positive cocci most likely has an enterococcal infection. Likewise, a gram-negative rod in a patient receiving an extended-spectrum penicillin would suggest a resistant coliform. The Gram's stain remains the cornerstone of rapid detection of intraamniotic infection in women with intact membranes and premature labor.[3,4] It has become the method of choice for defining bacterial vaginosis.[5] Protocols using this method of diagnosis have linked bacterial vaginosis to adverse pregnancy and gynecologic outcomes.

In this issue, Adriaanse and colleagues[6] report the rather poor performance of the Gram's stain in identifying group B streptococci in the lower genital tracts of parturients. However, they note that its diagnostic performance is comparable with the more expensive and technically advanced rapid antigen tests. This is valuable information, but it should not prevent us from using the Gram's stain in other more appropriate clinical scenarios. Its poor sensitivity is to be expected, given the threshold of $>10^4$ microorganisms per milliliter necessary to produce a positive test. Positive findings during the examination of clinical specimens remain a valid way to support the diagnosis of infection and direct antimicrobial therapy. We should also remember that the inexpensive cost of this test ($15 at our institution) makes it cost effective in most clinical circumstances. Christian Gram gave us a valuable tool that has proved its worth time and time again over the past century. We should not forget its utility in the care of women with infections.

*David E. Soper, Associate Editor*
*Department of Gynecology and Obstetrics*
*Medical College of Virginia*
*Richmond, Virginia*

### REFERENCES

1. Gram C: Ueber die Isolirte Farbung der Schizomyceten in Schnitt-und Trockenpraparaten. Forstschr Med 2:185–189, 1884.

Received September 13, 1995
Accepted September 13, 1995

Editorial

2. Centers for Disease Control (CDC): 1993 sexually transmitted treatment guidelines. MMWR 42:49, 1993.
3. Romero R, Yoon BH, Mazor M, Gomez R, et al.: A comparative study of the diagnostic performance of amniotic fluid glucose, white blood cell count, interleukin-6, and Gram stain in the detection of microbial invasion in patients with preterm premature rupture of the membranes. Am J Obstet Gynecol 169:839–851, 1993.
4. Romero R, Yoon BH, Mazor M, Gomez R, et al.: The diagnostic and prognostic value of amniotic fluid white blood cell count, glucose, interleukin-6, and Gram stain in patients with preterm labor and intact membranes. Am J Obstet Gynecol 169:805–816, 1993.
5. Nugent RP, Krohn MA, Hillier SL: Reliability of diagnosing bacterial vaginosis is improved by a standardized method of Gram stain interpretation. J Clin Microbiol 29:297–301, 1991.
6. Adriaanse AH, Muytjens HL, Kollée LAA, Nijhuis JG, Hoogkamp-Korstanje JAA: Significance of Gram's Stain in Rapid Intrapartum Screening for Maternal Carriership of Group B Streptococcus. Inf Dis Obstet Gynecol 3:110–115, 1995.
